# Bolivian natural zeolite as a low-cost adsorbent for the adsorption of cadmium: Isotherms and kinetics

**DOI:** 10.1016/j.heliyon.2024.e24006

**Published:** 2024-01-03

**Authors:** Lisbania Velarde, Dariush Nikjoo, Edwin Escalera, Farid Akhtar

**Affiliations:** a Department of Chemistry, Faculty of Science and Technology, Universidad Mayor de San Simón, UMSS, Cochabamba, Bolivia; b Division of Materials Science, Department of Engineering Sciences and Mathematics, Luleå University of Technology, SE-971 87, Luleå, Sweden

**Keywords:** Heavy metals, Natural zeolites, Cadmium, Adsorption, Clinoptilolite

## Abstract

Population growth in recent years has led to increased wastewater production and pollution of water resources. This situation also heavily affects Bolivia, so wastewater treatment methods and materials suitable for Bolivian society should be explored. This study investigated the natural Bolivian Zeolite (BZ) and its NaCl-modified structure (NaBZ) as adsorbents for cadmium removal from water. The natural BZ and the modified form NaBZ were investigated by different physicochemical characterization techniques. Furthermore, XPS and FT-IR techniques were used to investigate the adsorption mechanisms. The cadmium adsorption on BZ and NaBZ was analyzed using various mathematical models, and the Langmuir model provided a better description of the experimental adsorption data with cadmium adsorption capacities of 20.2 and 25.6 mg/g for BZ and NaBZ, respectively. The adsorption followed the pseudo-second order kinetics. The effect of different parameters, such as initial cadmium concentration and pH on the adsorption was studied. In addition, the results of the regeneration test indicated that both BZ and NaBZ can be regenerated by using hydrochloric acid (HCl). Finally, the adsorption experiment of BZ and NaBZ on a real water sample (brine from Salar de Uyuni salt flat) containing a mixture of different heavy metals was carried out. The results obtained in this study demonstrate the effectiveness of natural BZ and modified NaBZ in the removal of heavy metals from wastewater.

## Highlights

• Cd was successfully removed from the water by Bolivian natural zeolite (BZ).
• NaCl-treated Bolivian natural zeolite (NaBZ) showed higher removal of Cd.
• BZ and NaBZ showed ease of regeneration for cyclic use.
• Ion exchange and complexation mechanisms were suggested for the removal of Cd.

## Graphical abstract

**Figure:**
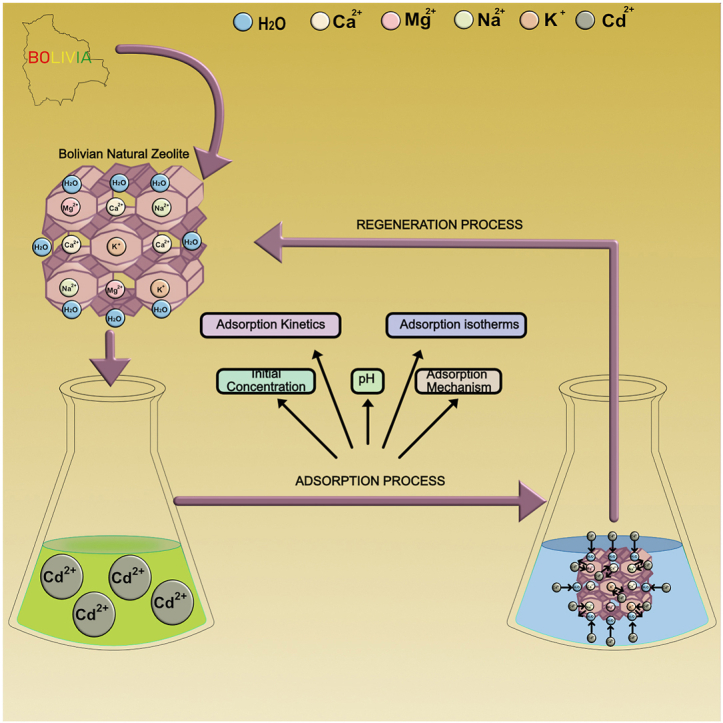

## 1 Introduction

Nowadays, water pollution is one of the major problems around the world. Removing toxic heavy metal ions from wastewater has attracted significant attention due to their harmful environmental and public health impact [[1], [2], [3]]. Cadmium is used in industry worldwide, and it is a common component in electric batteries, pigments, coatings, and electroplating and can contaminate the environment, especially water resources through its discharge to the wastewater [4,5]. Water contamination with cadmium above the permissible concentration limit is harmful because it is carcinogenic and can cause kidney, bone, and respiratory damage in humans [[6], [7], [8]].

According to the literature, many traditional approaches have been used to reduce cadmium concentrations or remove it from wastewater such as adsorption [[9], [10], [11]], bioremediation [12,13], coagulation [14], ion exchange, membrane separation [15], solvent extraction [16,17]. Amongst the above mentioned methods, adsorption is considered as a promising technique for cadmium removal due to its high efficiency, ease of use, low cost, and the availability of different adsorbents [1,18,19]. Natural zeolites are hydrated aluminosilicate porous materials a category of minerals with high availability and low cost compared to other adsorbents, offering good adsorption capacity for heavy metal ions due to their porous structure, accessible adsorption sites, and regeneration capabilities [[20], [21], [22]]. Furthermore, the pretreatment of natural zeolites with NaCl has been found significant for increasing the adsorption capacity of heavy metal removal from wastewater [23,24]. Additionally, recent studies demonstrate the potential for cadmium adsorption on natural zeolites from water, with removal efficiency between 70 and 99 % indicating that a large amount of cadmium concentration has been decreased after adsorption on zeolites [25,26]. The adsorption of heavy metals in natural zeolites can be facilitated through many mechanisms, including anion exchange, electrostatic attraction, intrapore diffusion, complexation, chemical reduction, etc. [[27], [28], [29]].

Bolivia is a country rich in mineralogical resources [18]. The availability of nonmetallic minerals is an excellent resource to use and convert these into specialized products [30]. The Bolivian natural zeolites as a natural mineral resource make them a suitable and advantageous choice for adsorbing heavy metals due to their unique properties, such as high surface area, ion exchange capacity, and regenerative capacity and being cost-effective and environmentally friendly [31]. Using local Bolivian natural resources can be highly beneficial for treating community water resources and industrial wastewater. In addition, studies on applying Bolivian natural zeolites for removing heavy metals in wastewater have not yet been reported. The results will be a significant antecedent to promote water treatment through natural resources, thus benefiting access to clean water in Bolivian communities.

The primary aims of this study are: i) modification of Bolivian natural zeolite (BZ) by treatment with NaCl (NaBZ), ii) physicochemical characterization of BZ and NaBZ by X-ray diffractometer (XRD), Scanning Electron Microscopy (SEM), and N_2_ adsorption-desorption isotherms, iii) application of BZ and NaBZ for the adsorption of cadmium in water through batch experiments, iv) study of the adsorption isotherms and kinetics, v) analysis of the pH and initial concentration parameters, vi) analyze the possible mechanisms by X-ray photoelectron spectroscopy (XPS) and Fourier transform infrared spectroscopy (FT-IR), vii) evaluation of the regeneration of BZ and NaBZ, viii) analysis of water samples from the Salar de Uyuni brine in Bolivia, which contain Cd (1.35 mg/L) along with As (6.74 mg/L), Sb (0.36 mg/L), Cu (16.03 mg/L), Co (11.3 mg/L), Fe (398.6 mg/L), Li (265.3 mg/L), Mg (5124 mg/L), Mn (3.94 mg/L), Ni (36.52 mg/L), Pb (9.53 mg/L), Na (127177.6 mg/L), K (3000 mg/L), Se (407 mg/L), and Zn (235 mg/L) to verify the feasibility of using BZ and NaBZ adsorbents for the removal of heavy metal admixture in the solutions. The study demonstrates the potential of clinoptilolite, a natural zeolite of Bolivian origin, for removing heavy metals from water.

## 2 Materials and methods

### 2.1 Materials

Natural Bolivian Zeolite (BZ) of clinoptilolite type was obtained from Sucre, Bolivia. First, BZ was crushed and sieved in the 45–500 μm range, then washed with abundant distilled water and dried for 24 h at 105 °C. The stock Cd solution was prepared by dissolving CdCl_2_ in distilled water. Solutions of 0.1 M and 0.5 M of HCl and NaOH were used to adjust the pH. NaCl and AgNO_3_ were used for the conversion of BZ into a Na-form and followed by verification of the elimination of chlorine ions (Cl^−^), respectively. All reagents used were of analytical grade and were purchased from Merck KGaA, Darmstadt, Germany.

The brine sample to be processed was acquired from the Salar de Uyuni salt flat in Potosi, Bolivia, in September 2019 (geographical coordinates: 20° 17′ 22″ S 67°04′35″ W).

### 2.2 Bolivian natural zeolite pretreatment

The modification treatment to convert BZ in Na-form was prepared according to previous procedures described in the literature [32,33]. 100 g of BZ was added to a 2 M NaCl solution, and the solution was stirred at 120 rpm and 25 °C for 24 h, then filtered and washed with copious amounts of distilled water to remove the Cl^−^ completely. The resultant pretreated zeolite was designated as NaBZ. The presence of Cl^−^ was verified using the silver nitrate (AgNO_3_) (0.1 M) assay to ensure that the Cl^−^ was removed. In the mentioned method, a zeolite sample was first placed in distilled water, and the solution was acidified with a few drops of HNO_3_ (0.1 %) (pH = 4.8) to remove carbonates, which can form a white residue that can be mistaken for the precipitates of Cl^−^. Then, a few drops of AgNO_3_ were slowly added to the solution. This step was essential to observe whether a white precipitate formed after adding AgNO_3_ to confirm or rule out the presence of Cl^−^ ions.

### 2.3 Characterization of zeolites

The crystallinity of BZ and NaBZ was determined by X-ray powder diffraction (XRD) using an ADP 2000 Pro X-ray diffractometer (Italy) with Cukα radiation (λ = 1.5418 Å) in the 2Θ range of 5–50 with a step of 0.02. The microstructure was characterized by a scanning electron microscope (SEM, JSM-IT300LV, JEOL GmbH, German). Textural properties were measured at −196 °C using Gemini VII 2390, Micromeritics, Norcross, GA, USA. The characterization of BZ and NaBZ before and after the adsorption of cadmium by Fourier transform infrared spectroscopy (FT-IR, Vertex 70v vacuum-based, USA), and X-ray photoelectron spectroscopy (XPS) (Kratos Analytical Ltd., UK) were used to investigate the mechanism.

### 2.4 Batch adsorption experiments

The adsorption of cadmium on BZ and NaBZ was carried out by batch method. 1 g of zeolite was added to 50 mL of 500 mg/L of Cd. The solution was stirred at 200 rpm for 24 h. Once equilibrium was achieved, the solution was filtered with a syringe filter of 0.45 μm to collect the final solutions. The difference between initial and final metal concentrations in the solution determined cadmium adsorption. The initial and final concentrations of cadmium solutions were measured by inductively coupled plasma sector field mass spectrometry (ICP-SFMS, USA) by a certified analytical laboratory (ALS Scandinavia, Sweden). The following equation calculated the adsorption capacity of the BZ and NaBZ at equilibrium:

(1) $$qe=\left(C_{0}-C_{e}\right)V/W$$

where C_0_ and C_e_ are the initial and equilibrium concentrations of Cd in the solution (mg/L), V is the volume of solution (L), and W is the weight of adsorbent (g).

The removal efficiency was calculated as follows:

(2) $$R=\left(C_{0}-C_{e}\right)/C_{0}\times 100\%$$

where C_0_ and Ce are the initial and equilibrium of cadmium concentration in the solution (mg/L).

**Fig. 1:**
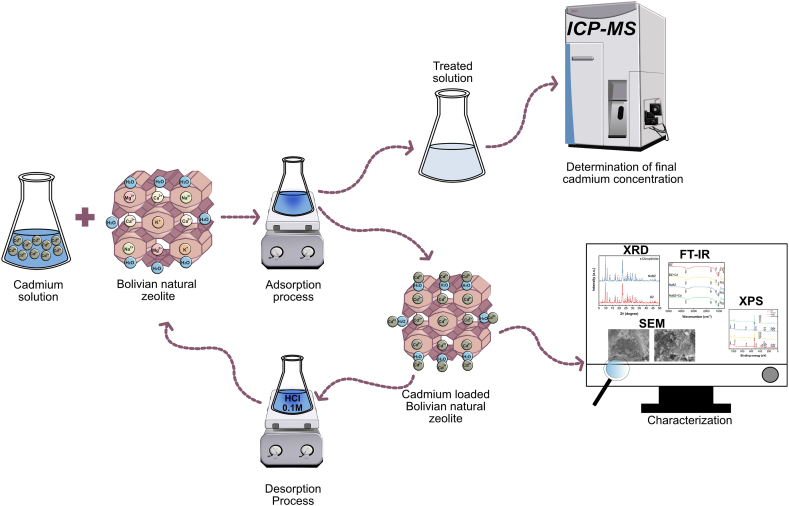
Adsorption and regeneration processes of Cd on raw (BZ) and Na-form Bolivian natural zeolite (NaBZ).

The adsorption isotherms and kinetics were obtained, varying the initial cadmium concentration in the range of 10–500 mg/L over 5–180 min. Additionally, the effect of pH (5.5, 6, 7, and 11) were studied with other parameters being constant (C_0_ = 500 mg/L, pH = 6, and 1 g of natural zeolite). Fig. 1 illustrates the methodology employed for the adsorption and regeneration of natural BZ.

#### 2.4.1 Adsorption isotherms

Adsorption measurements were performed at different initial concentrations ranging from 10 to 500 mg/L. The experimental data were analyzed using Langmuir and Freundlich isotherm models [34,35] to investigate and describe the adsorption behavior of cadmium on BZ and NaBZ.

The following equations describe the Langmuir and Freundlich model:

The following equation represents the Langmuir model:

(3) $$q_{e}=k_{L}q_{m}C_{e}/\left(1+k_{L}C_{e}\right)$$

The linear form of the Langmuir model is:

(4) $$C_{e}/q_{e}=1/q_{m}k_{L}+\left(1/n\right)C_{e}$$

where q_e_ is the equilibrium adsorption, and q_m_ is the maximum adsorption capacity (mg/g), C_e_ is the equilibrium concentration (mg/L) related, and k_L_ is the Langmuir isotherm constant (L/mg) related to the affinity of the binding sites to the adsorbate.

The following equation represents Freundlich model:

(5) $$q_{e}=k_{F}{C_{e}}^{1/n}$$

The Freundlich model in the linear form is:

(6) $$\ln\;q_{e}=\ln\;k_{F}+\left(1/n\right)\ln\;C_{e}$$

where q_e_ is the equilibrium adsorption (mg/g), C_e_ is the equilibrium concentration (mg/L), k_F_ is the Freundlich isotherm constant (mg/g, and 1/n (dimensionless) represents the heterogeneity of the adsorbent sites and indicates the affinity between adsorbate and adsorbent.

#### 2.4.2 Adsorption kinetics

The adsorption kinetics of Cd on BZ and NaBZ were studied, fitting the experimental data with the pseudo-first order and pseudo-second order kinetics models (Ho & McKay, 1998). The pseudo-first order equation determines the adsorption rate according to the amount of metal adsorbed versus time t as follows:

(7) $${dq}_{t}/d_{t}=k_{1}\left(q_{e}-q_{t}\right)$$

In the linear form, the pseudo-first-order equation is expressed by:

(8) $$\log\;\;\log\;\left(q_{e}-q_{t}\right)=\log\;\;\log\;q_{e}\;-k_{1}/2.303\times t$$

where q_e_ and q_t_ are the amounts of metal ions adsorbed (mg/g) at time t (min) and at equilibrium, respectively, k_1_ (1/min) is the rate constant of adsorption.

The following equation describes pseudo-second order:

(9) $${dq}_{t}/d_{t}=k_{2}{\left(q_{e}-q_{t}\right)}^{2}$$

The transformation in the linear form is represented as follows:

(10) $$t/q_{t}=1/k_{2}\;{q_{e}}^{2}+t/q_{e}$$

where k_2_ is the pseudo-second-order rate constant (g.mg/min), q_e_ and q_t_ (mg/g) are the metal ions adsorbed at equilibrium and time t, respectively.

### 2.5 Regeneration process

The regeneration process (see Fig. 1) was performed by adding a cadmium saturated BZ and NaBZ in a desorbing solution of 0.1 M HCl solution at room temperature and 24 h. After the regeneration process, the samples were washed with distilled water to remove the excess of Cl^−^ ions. The regeneration process was performed three times to verify the regeneration performance of BZ and NaBZ.

### 2.6 Adsorption of heavy metals from Salar de Uyuni

The adsorption of heavy metals from the brine of Salar de Uyuni salt flat on BZ and NaBZ was carried out similarly as described in section 2.3. 1 g of zeolite was added to 50 mL of brine, and the solutions were stirred at 200 rpm, pH 6, and 25 °C for 24 h. After adsorption, the samples were filtered using a 0.45 μm syringe filter to recover BZ and NaBZ and measure the final concentrations using an atomic absorption spectrophotometer.

The equilibrium adsorption, kinetics, and regeneration measurements were repeated three times, and the average of three measurements was reported with the error bars.

## 3 Results and discussion

### 3.1 Zeolite characterization

**Fig. 2:**
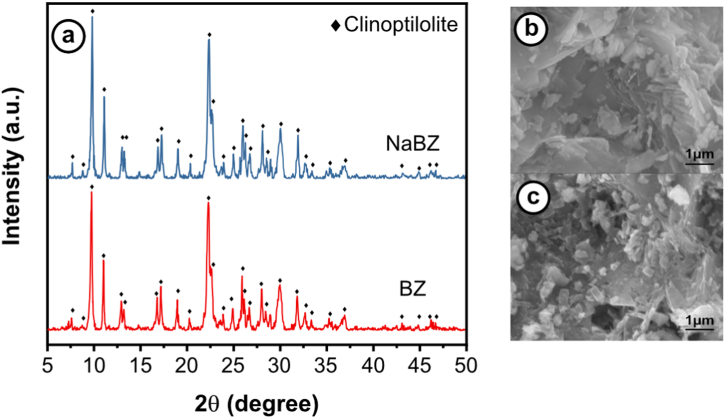
a) X-ray diffractograms of BZ and NaBZ, **b)** SEM image of BZ, and c) SEM image NaBZ.

The X-ray diffractograms of BZ and NaBZ in Fig. 2a show that the main diffraction peaks of BZ appears at 2θ of 9.90°, 11.20°, 17.38°, 22.45°, 28.18°, 30.34°, and 32.03° corresponding to hkl plans of $\left(020\right),\left(200\right),\left(111\right),\left(131\right),\left(\bar{4}22\right),\left(151\right),\left(\bar{2}61\right)$, respectively. These diffraction peaks are characteristic of clinoptilolite type structure, in agreement with the diffraction pattern described by Treacy [36]. It can be observed in Fig. 2a that there was no significant change in the BZ crystal structure after treatment by NaCl, suggesting the structural stability of the clinoptilolite during the modification. This observation is consistent with previous studies that also reported no degradation of the crystalline structure of natural zeolites due to the treatment with NaCl [37,38].

SEM images of BZ (Fig. 2b), and NaBZ (Fig. 2c) show similar stacked flake structures. The morphology of the BZ did not change significantly after treatment with NaCl, as previously reported [37,39,40]. Both BZ and NaBZ exhibit a stacked flake structure peculiar to clinoptilolite, as previously reported, and the presence of fine particles in the surface of the zeolitic material can be observed, which can confirm the modification of BZ as was reported in previous studies [41,42].

The surface area and porosity of BZ and NaBZ were measured by N_2_ adsorption–desorption isotherms at −196 °C, as depicted in Table 1. The results show a slight increase in the surface area after the treatment with NaCl. These results are consistent with those published in the literature. Bektaş and Kara [32] reported an increase in the surface area of natural zeolite from 15.36 to 16.41 m^2^/g after treatment with NaCl. Also, Gedik and Imamoglu (2008) reported 36.7 and 40.4 m^2^/g values for natural and NaCl-treated clinoptilolite, respectively. The slight increase in the surface area could be due to the removal of impurities such as quartz and feldspar from BZ during NaCl treatment [[43], [44], [45]]. It seems both that the increase in surface area and pore volume were effective for enhancing the adsorption of Cd on NaBZ compared to BZ. Likewise, after treatment of BZ in NaCl, an increase in micropore area (9.41 m^2^ g-16.89 m^2^/g) and pore diameter (13.88 Å −15.31 Å) were evidenced, revealing the advantage of NaBZ in Cd adsorption [46].

**Table 1:** Textural parameters of Bolivian natural zeolite (BZ) and Na-modified (NaBZ).

Parameter	Sample	
	BZ	NaBZ
**Surface Area (m**^**2**^**/g)**	25.93	30.94
**Pore Diameter (Å)**	13.88	15.31
**Pore Volume (cm**^**3**^**/g)×10**^**−**^**^3^**	5.14	9.16
**Micropore area (m**^**2**^**/g)**	9.41	16.89

**Fig. 3:**
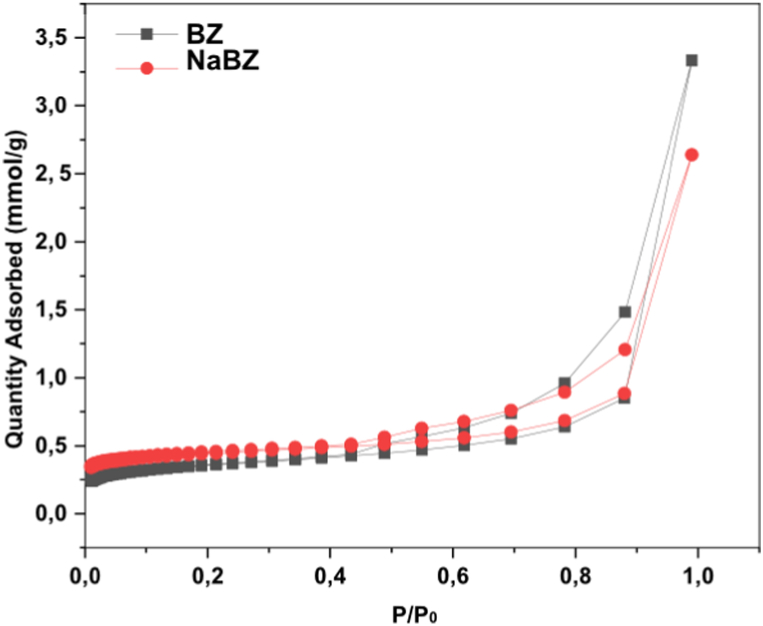
N_2_ adsorption-desorption isotherms of BZ and NaBZ.

Furthermore, according to Fig. 3., it can be observed that the N_2_ adsorption-desorption isotherms on BZ and NaBZ show an isotherm type IV according to IUPAC and present hysteresis loops type H1, indicating that BZ and NaBZ are mesoporous minerals consisting of agglomerations of uniform spheres in a regular shape with narrow pore size distribution [[47], [48], [49]].

### 3.2 Batch adsorption test

#### 3.2.1 Effect of initial concentration and pH

**Fig. 4:**
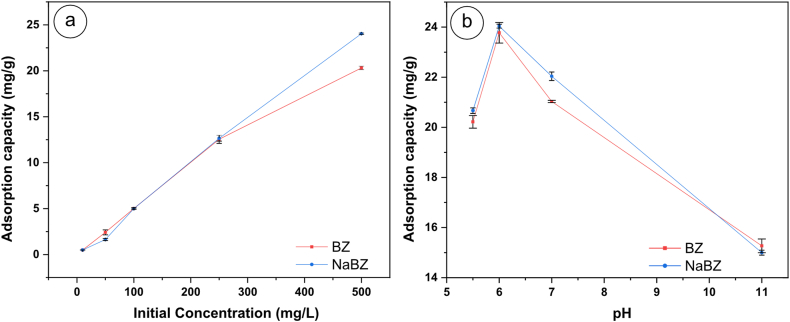
Effect of **a)** initial concentration, and **b)** pH of Cd adsorption in BZ, and NaBZ.

As depicted in Fig. 4a, the adsorption capacity of Cd rises proportionally with the initial concentration. Such a phenomenon can be attributed to a more significant amount of Cd in the solution, which implies the increase in driving force for the mass transfer of Cd to the surface of BZ and NaBZ [[50], [51], [52]].

Furthermore, the pH parameter was also examined, as it is crucial for understanding the behavior of Cd in the adsorption process. Fig. 4b illustrates that the adsorption capacity initially increases with an increase in pH from 5.5 to 6. This occurs because higher pH values reduce the presence of hydrogen ions, thereby providing more sites for Cd adsorption. Subsequently, an increase in pH from 6 results in a decrease in Cd adsorption due to the formation of Cd(OH)_2_, in line with the findings of Khan et al. [53] and S. Wang et al. [54]. Also, the results agree with the cadmium Pourbaix diagram, where it shows that with pH values lower than 7 the dominant species is Cd^2+,^ and at higher pH values, Cd starts to precipitate and appear in the form of Cd(OH)_2_ [[55], [56], [57], [58]]**.**

#### 3.2.2 Adsorption isotherms

**Fig. 5:**
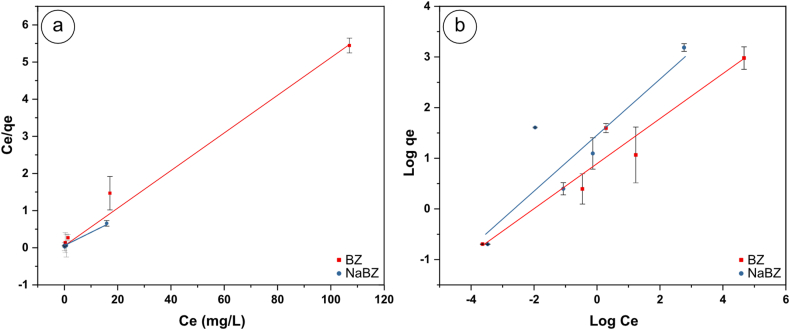
a) Langmuir and **b)** Freundlich isotherms of Cd adsorption in BZ and NaBZ.

Fig. 5 shows the linear plots of Cd on BZ and NaBZ, respectively, using the Langmuir and Freundlich models. Also, Table 2 presents the adsorption parameters, which show that Cd adsorption on both BZ and NaBZ fits the Langmuir adsorption model with correlation factors (R^2^) of nearly 1. Therefore, the adsorption of Cd on BZ and NaBZ was best described by the Langmuir model, which showed single-layer adsorption of Cd on a homogeneous surface of BZ and NaBZ.

**Table 2:** Langmuir and Freundlich isotherms parameters of Cd adsorption in BZ and NaBZ.

Sample	Langmuir			Freundlich		
	q_max_ (mg/g)	K_L_ (L/mg)	R^2^	n (L/mg)	K_F_ (mg/g)	R^2^
**BZ**	20.28	0.20	0.99	2.27	2.42	0.92
**NaBZ**	25.64	1.07	0.99	1.83	4.66	0.77

The maximum adsorption capacity (q_m_) and removal efficiency for BZ and NaBZ were 20.2 (78.6 %) and 26.5 (96.9 %) mg/g, respectively. The obtained results showed higher cadmium adsorption in comparison to some studies that reported lower Cd adsorption on different natural zeolites from around the world, such as Ukrainian clinoptilolite (4.22 mg/g) [59], Iranian clinoptilolite (4.00 mg/g) [60], Australian clinoptilolite (1.32 mg/g) [61].

In addition, the Freundlich adsorption parameters show that the K_F_ value is higher in Cd adsorption on NaBZ than on BZ, indicating that the maximum amount of adsorption is higher on NaBZ than on BZ [52]. Additionally, the parameter of 1/n and K_L_ indicates the adsorption intensity, if the values of 1/n are less than 1 the adsorption is favorable. The values of 1/n for cadmium adsorption on BZ and NaBZ are 0.44 and 0.55, respectively, thus determining the favorability of the Cd adsorption process on BZ and NaBZ [50,[62], [63], [64]]. While making direct comparisons with literature on cadmium adsorption, the difficulty is inherited due to different experimental conditions and sources of chemicals used.

#### 3.2.3 Adsorption kinetics

**Fig. 6:**
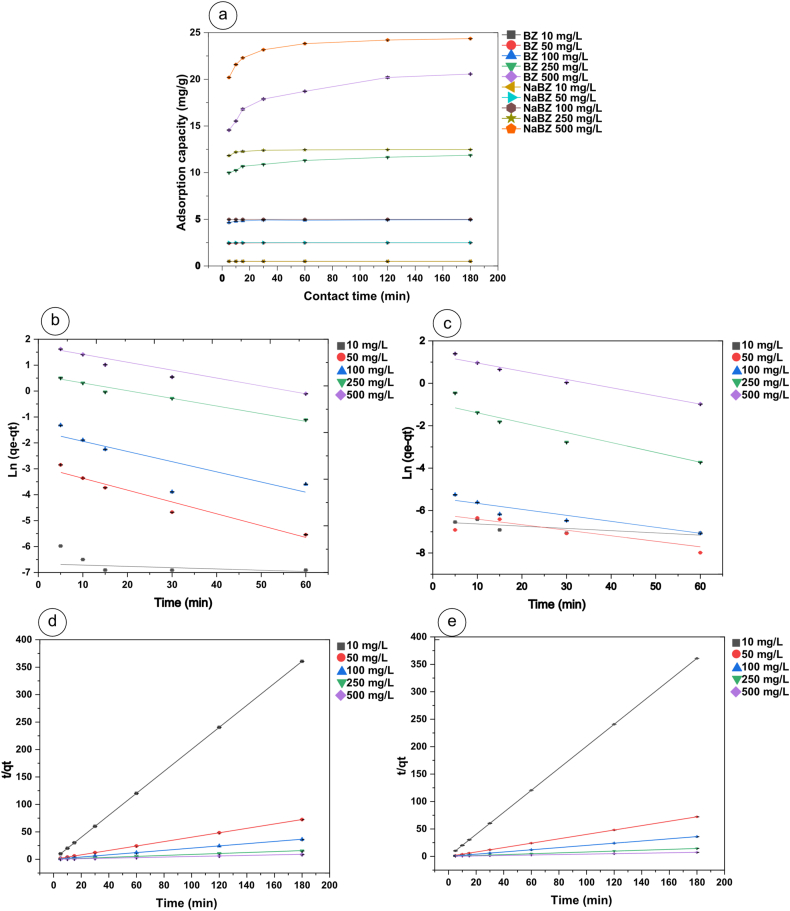
a) Effect of contact time between Cd adsorption on BZ and NaBZ, **b)**, Pseudo-first order adsorption kinetic of Cd on BZ, **c)** Pseudo-first order adsorption kinetic of Cd on NaBZ, **d)** Pseudo-second order kinetic of Cd on BZ, **e)** Pseudo-second order kinetic of Cd on NaBZ.

First, the adsorption capacity of Cd on BZ and NaBZ for different initial concentrations was plotted against time to verify the equilibrium time. According to Fig. 6a, the uptake of Cd reached the equilibrium at 60 min. An equilibrium time of 120 min was selected for further analysis.

The experimental data were fitted using a linear form of the adsorption kinetics models of pseudo-first order (equation (8)) and pseudo-second order (equation (10)). The fitting results will provide additional information on the adsorption mechanism of Cd on BZ and NaBZ. The fit of the data may indicate whether the adsorption involves a rate-limited control step if it conforms to first-order kinetics, however, the data confirms to second-order kinetics the adsorption mechanism is chemical [29,65,66].

The sorption kinetic process generally consists of bulk diffusion from the solution to the adsorbent, diffusion of metal ions through the boundary layer, and sorption at the adsorption site. However, in our case, the agitation in the experimental procedure may influence the bulk diffusion process. According to the data fitting results of Fig. 6b-e, the pseudo-second-order kinetic model with a correlation factor (R^2^) greater than 0.99 explained the experimental data better. Moreover, the observed values of qe_exp_ were similar to those of q_cal_, as shown in Table 3. Hence, it was evidenced that the adsorption process of Cd on BZ and NaBZ follows the pseudo-second order kinetic which indicates a chemisorption [[67], [68], [69]]. The result is consistent with fitting the data to the Langmuir isotherm, which is a monolayer, as chemisorption has been reported to occur in a monolayer [70].

**Table 3:** Comparison of the first and second order kinetics of Cd adsorption in Bolivian natural zeolite.

Adsorbent	Initial Cd concentration (mg/L)	qe_exp_ (mg/g)	Pseudo first order			Pseudo second order		
			K_1_ (1/min)	qe_cal_ (mg/g)	R^2^	K_2_ (g/mg min)	q_cal_ (mg/g)	R^2^
**BZ**	10	0.499	0.011	0.001	0.412	2.003	0.499	1
	50	2.482	0.046	0.054	0.943	2.237	2.48	1
	100	4.93	0.041	0.202	0.677	0.529	4.95	1
	250	11.64	0.028	1.734	0.975	0.039	11.93	0.999
	500	19.65	0.030	5.090	0.950	0.012	20.74	0.999
**NaBZ**	10	0.49	0.010	0.001	0.573	36.557	0.498	1
	50	2.49	0.026	0.001	0.788	48.654	2.49	1
	100	4.99	0.030	0.004	0.881	13.36	4.99	1
	250	12.46	0.054	2.070	0.902	0.329	12.48	1
	500	24.2	0.041	4.054	0.976	0.027	24.50	1

The short time in which Cd adsorption reached equilibrium showed the effectiveness and efficiency of BZ and NaBZ to adsorb Cd as a model heavy metal pollutant.

#### 3.2.4 Regeneration process

**Fig. 7:**
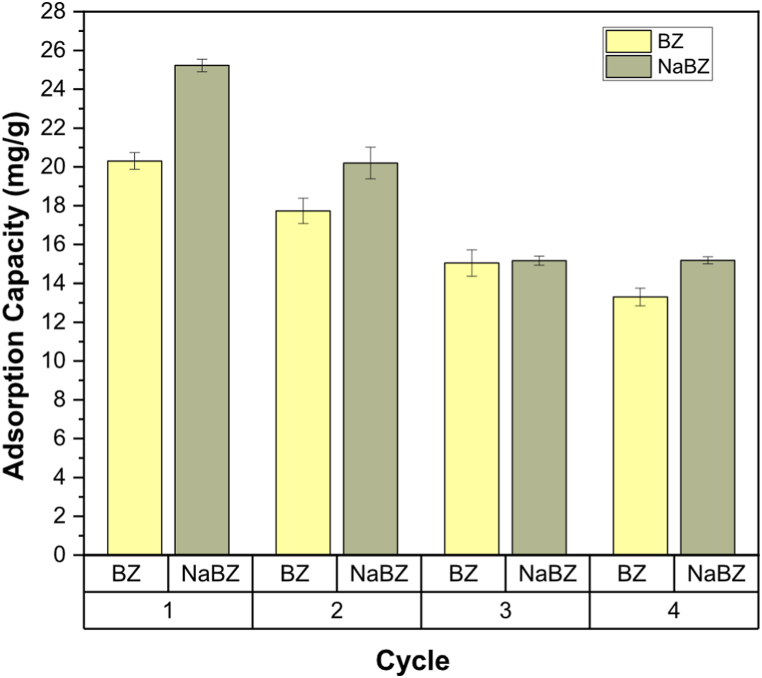
Adsorption cycles of Cd in BZ and NaBZ.

The regeneration process was crucial in studying BZ and NaBZ efficiency, as it allows for investigating their pilot scale application. The analysis of kinetics and isotherms developed in sections 3.2.1. and 3.2.2. showed that the adsorption of Cd on BZ and NaBZ occurs by chemisorption. Therefore, the binding between Cd and zeolites is strong, suggesting an irreversible reaction [60]. This assumption was confirmed in the regeneration process as the amount of metal desorbed is less than the adsorbed concentration, indicating that some amount of Cd remains on BZ and NaBZ after desorption, causing a decrease in adsorption capacity after each cycle, as shown in Fig. 7.

In this manner, the regenerative capacity of BZ and NaBZ have been verified and they are considered as potential efficient and environmentally friendly adsorbents.

#### 3.2.5 Adsorption mechanism

**Fig. 8:**
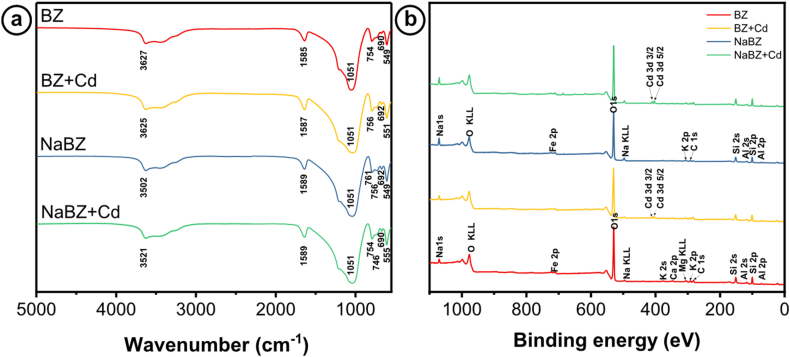
a) FT-IR spectra, and **b)** XPS spectra of BZ and NaBZ before and after Cd adsorption.

FT-IR and XPS techniques were employed to investigate the adsorption mechanism of Cd in BZ and NaBZ. Fig. 8a shows no difference between the FT-IR spectra of BZ and NaBZ. However, following the adsorption of Cd, a shift in the characteristic hydroxyl wavenumbers (3700 cm^−1^ and 1600 cm^−1^) confirmed an interaction between Cd and hydroxyl groups [71]. In addition, the FT-IR spectra indicated the presence of characteristic peaks of clinoptilolite, including the asymmetrical vibration of Si–*O*–Si at 1051 cm^−1^, bending vibrations of Si–*O*–Si (690-692 cm^−1^), and stretching Si–O at (754-761 cm^−1^^)^ [72,73].

Using XPS, the elemental composition was determined, and the results in Fig. 8b confirmed the adsorption of Cd^2+^ on BZ and NaBZ. This was suggested by the appearance of two peaks at 406.8 eV and 413.5 eV, which are characteristic of Cd 3d^3/2^ and Cd 3d^5/2^, respectively [74,75]. Moreover, a reduction in the content of exchangeable cations of zeolite (Na, Ca, and K) was observed after Cd adsorption, suggesting an ion exchange mechanism [67,76]. Additionally, the decrease in O groups indicated an interaction between hydroxyl groups and Cd [77,78]. Therefore, based on the results, it can be concluded that the adsorption mechanism of Cd on BZ and NaBZ involves ionic exchange and complexation with hydroxyl groups.

**Fig. 9:**
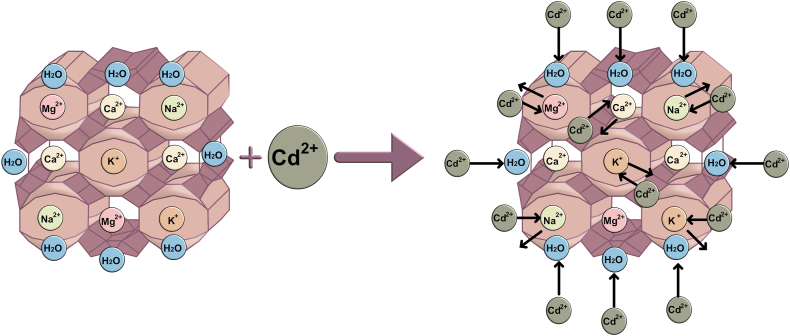
Adsorption mechanism of Cd on natural BZ.

A schematic representation of the hypothetical mechanism of Cd adsorption on BZ is presented in Fig. 9.

### 3.3 Adsorption of heavy metals from Salar de Uyuni

**Fig. 10:**
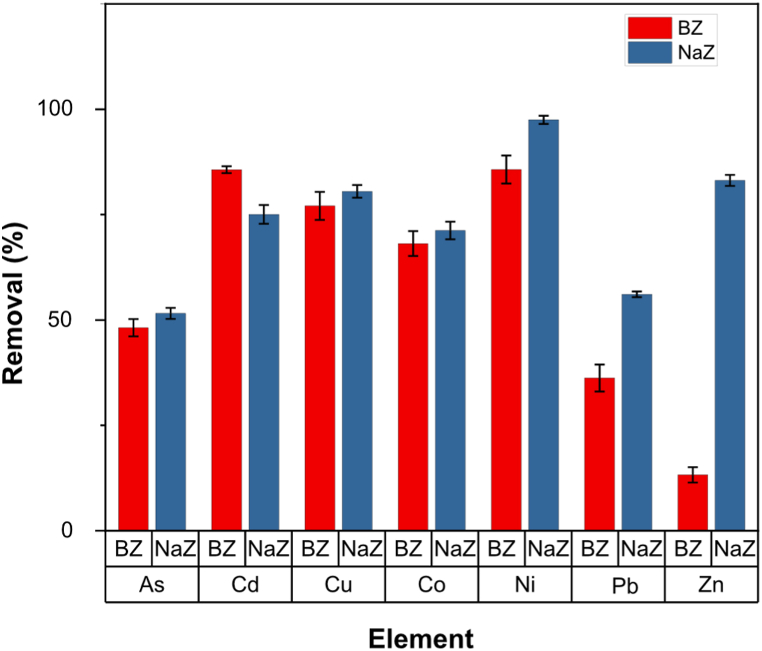
Adsorption of heavy metals from the brine of Uyuni salt flat on BZ and NaBZ.

Fig. 10 illustrates that using BZ and NaBZ proved to be effective in removing various heavy metals. Cd was found to be strongly adsorbed by BZ, while NaBZ had a high affinity for As, Cu, Co, Ni, Pb, and Zn. The removal efficiencies for BZ were as follows: As (46 %), Cd (84.8 %), Cu (73.6 %), Co (67.5 %), Ni (85.7 %), Pb (33.5 %), and Zn (12.4 %). The corresponding removal efficiencies for NaBZ were: As (51.9 %), Cd (73.3 %), Cu (79 %), Co (69.4 %), Ni (96.6 %), Pb (55.9 %), and Zn (81.8 %). The results indicate that using NaCl to treat BZ resulted in the increased removal efficiencies for most elements, except for Cd, which, although it did not have an increase in removal efficiency, obtained a favorable adsorption value over other heavy metals. The non-increase in cadmium removal efficiency on NaBZ may be attributed to the higher affinity of Na cations towards the other elements. These findings are consistent with previous studies, which have reported an increase in adsorption after NaCl treatment [79].

The selectivity of BZ and NaBZ were Ni > Cd > Cu > Co > As > Pb > Zn and Ni > Zn > Cu > Cd > Co > Pb > As, respectively. Contrary to the reported in the literature, BZ and NaBZ show high selectivity for Ni and low selectivity for Pb [67,69,80]. This could be due to the higher quantity of Ni than Pb in the brine from Salar de Uyuni. The obtained results might be suitable for using the treated brine as a suitable grown medium of the microorganism Halomonas boliviensis [81]. Since the quantity of heavy metals has decreased, the toxicity in the culture medium will be lower, thus providing a more optimal culture medium for the mentioned microorganism. Likewise, the hypothesis of the study was verified. Bolivian natural zeolite is suitable for the adsorption of cadmium and other heavy metals from the brine of the Salar de Uyuni, which is a complex real sample due to many elements in its composition.

The investigation of utilizing natural zeolites for adsorbing pollutants is a commonly conducted research due to its significant environmental impact. To date, there is a lack of reported studies regarding the viability of Bolivian natural materials, such as natural zeolites, for their potential use in the adsorption of pollutants in synthetic and actual water samples. Furthermore, it has been observed that Bolivian natural zeolite exhibits a greater affinity for cadmium adsorption than natural zeolites sourced from alternative geographical locations. Moreover, it presents a promising solution to address global pollution and drought.

Nevertheless, it is important to acknowledge several limitations inherent in this study. For instance, one such constraint pertains to the duration required for executing experimental tests on a wide scale. The study also encounters challenges related to the intricate nature of interactions within mixed media.

## 4 Conclusions

According to the obtained results, the adsorption of Cd is effective on raw and NaCl-treated Bolivian natural zeolite with adsorption values of 20.2 and 25.6 mg/g, respectively. Also, the adsorption isotherms of Langmuir and Freundlich and the pseudo-first and pseudo second order adsorption kinetics were studied. The results revealed that the Langmuir isotherm and pseudo-second order kinetic model describes the experimental Cd adsorption and kinetic data on BZ and NaBZ. The desorption showed the regeneration and reusability of the Bolivian natural zeolite. The characterization of BZ and NaBZ before and after adsorption revealed ionic exchange and interaction with hydroxyl groups as the main mechanisms of the adsorption process of Cd on BZ and NaBZ. Moreover, the adsorption of Cd and other heavy metals (As, Cu, Co, Ni, Pb, Zn) from a real water sample such as the brine of Salar de Uyuni demonstrated the adsorption capacity of BZ and NaBZ.

Without a doubt, the current study provides further evidence about the adsorption capabilities of natural zeolites and their potential applicability in the development of water treatment systems. Furthermore, this study presents a framework for future investigations into the alteration and application of Bolivian natural zeolite in the adsorption of diverse inorganic and organic contaminants originating from various sources of pollution. Furthermore, it is imperative to prioritize the desorption method, conduct thorough analysis of water samples obtained from real sources, and ensure the application of the water purification system for the purpose of safeguarding public health and environmental protection.

## Funding

Swedish International Development Cooperation Agency (SIDA), contribution No. 13486.

## Data availability

Data will be made available on request.

## CRediT authorship contribution statement

**Lisbania Velarde:** Writing - original draft, Investigation, Formal analysis, Data curation, Conceptualization. **Dariush Nikjoo:** Writing - review & editing, Formal analysis. **Edwin Escalera:** Writing - review & editing, Project administration, Funding acquisition, Conceptualization. **Farid Akhtar:** Writing - review & editing, Supervision, Resources, Project administration, Funding acquisition, Formal analysis, Conceptualization.

## Declaration of competing interest

The authors declare that they have no known competing financial interests or personal relationships that could have appeared to influence the work reported in this paper.
